# Consideraciones acerca del uso de glucómetros durante la prueba de tolerancia oral a la glucosa

**DOI:** 10.1515/almed-2024-0108

**Published:** 2024-12-17

**Authors:** Bernardo Alio Lavin-Gomez, Armando Raúl Guerra Ruíz

**Affiliations:** 16516 Hospital Universitario Marques de Valdecilla Ringgold Standard Institution – Analisis Clinicos Santander, Cantabria, España

**Keywords:** diabetes mellitus, prueba de tolerancia oral a la glucosa, pruebas a la cabecera del paciente

Estimado Editor,

Hemos leído con gran interés el artículo de Fabre-Estremera y colaboradores [1], y les felicitamos por su publicación que aborda un tema importante para el laboratorio clínico. El artículo realiza un estudio comparativo entre glucómetros-POCT de uso profesional y el laboratorio central durante la prueba de tolerancia oral a la glucosa (PTOG) para el diagnóstico de prediabetes y diabetes, y evalúa la conectividad de los mismos a la historia clínica del paciente y la reducción de costes. La glucosa es un parámetro fundamental en el diagnóstico y seguimiento de los pacientes diabéticos o con intolerancia a la glucosa. El rigor en su determinación en cualquier situación ha de ser un punto primordial de la actividad clínica, tanto intrahospitalaria como extra-hospitalaria. Sin embargo, muchas empresas proveedoras de glucómetros se han enfocado al autocontrol del paciente diabético, y no a la determinación de la glucemia de uso profesional. Nos gustaría destacar algunas consideraciones críticas que podrían aportar al debate sobre el tema.

1. Manejo preanalítico de las muestras: Nos llama la atención que para evitar la glucólisis en las pruebas de sobrecarga, no se hayan utilizado contenedores con inhibidor de la misma en todos los tiempos. Según las recomendaciones actuales para minimizar la glucólisis en las muestras de sangre se debe utilizar un tubo que contenga un inhibidor glucolítico de efectividad rápida. En caso de no ser posible, el tubo con la muestra debe colocarse inmediatamente en agua y hielo y centrifugarse en los primeros 30 minutos posteriores a la recolección [2]. La omisión de esta consideración podría llevar a una subestimación de los niveles reales de glucosa en sangre debido a la glucólisis post-recolección, afectando así la precisión de la comparación con los glucómetros evaluados.
2. Correlación entre las determinaciones de glucosa: Los autores encuentran diferencias estadísticamente significativas entre las concentraciones de glucosa medidas en ayunas, siendo los glucómetros los que mostraron concentraciones mayores. Aunque las consideraciones preanalíticas señaladas en el punto anterior podrían ser la explicación de este hallazgo, y así reflexionan los autores en la discusión, llama la atención el comportamiento del total de las muestras en la regresión. Al observar las gráficas de regresión y Passing-Bablok en el Material Suplementario parece haber una tendencia clara en las diferencias, que aumentan significativamente a la par que los valores de glucosa del laboratorio central. Esto parece indicar un sesgo sistemático hacia los valores altos, donde los valores medidos con POCT son inferiores al del laboratorio. Nos preguntamos si los autores tienen alguna hipótesis al respecto.
3. Por último, pero no por ello menos importante, varios estudios han encontrado que niveles anormales de hematocrito pueden interferir con las lecturas de glucosa de los glucómetros [3], [4], [5]. En general, niveles bajos de hematocrito tienden a resultar en lecturas de glucosa artificialmente elevadas, mientras que niveles altos pueden causar lecturas más bajas. Para pacientes pediátricos como los de este trabajo, es recomendable establecer rangos de referencia diferentes para el hematocrito por edad y sexo, que abarcarían desde un valor más bajo de 34 % hasta el valor más alto de 54 %, por lo que las diferencias pueden resultar significativas [6]. No obstante, la mayoría de los glucómetros considera para los cálculos un hematocrito invariable fijado en un 43 % según recomendación IFFC [7]. La misma guía recomienda un factor de conversión automático entre plasma y sangre total fijado en el 11 % (F=1,11), que es el generalmente implementado por los fabricantes.

Según nuestra experiencia (datos no publicados), un valor de glucemia en muestras heparinizadas, plasma en laboratorio central (Atellica^®^Solution-CH; Siemens-Healthineers, Erlangen, Alemania), método enzimático (glucosa hexoquinasa), frente a sangre total en un glucómetro de uso profesional que no ajusta el resultado según hematocrito real del paciente, y otro también disponible comercialmente que mide el hematocrito de forma concomitante y realiza una conversión individual para cada muestra, mostró los resultados expuestos en la Tabla 1. En ellos se aprecia que la ausencia de ajuste por hematocrito real conlleva una tendencia marcada a emitir valores más elevados con hematocritos más bajos y viceversa (incrementos hasta 23 % y disminuciones de hasta 19 %, respectivamente) a pesar del factor de conversión automático. Ambos glucómetros cumplían con las recomendaciones de la ISO 15197:2013 (al menos el 95 % de los valores dentro del ±15 mg/dL para glucemias <100 mg/dL, y ±15 % para glucemias ≥100 mg/dL).

**Tabla 1:** Concentraciones de Glucosa (mg/dL) en plasma heparinizado en laboratorio central (método: glucosa hexoquinasa, Atellica^®^ Solution-CH; Siemens-Healthineers) Vs. concentraciones de glucosa en sangre total heparinizada medida en Glucómetros- POCT.

Hematocrito, %	Glucosa, mg/dL, media (CV%)		
(rango)	Lab central	Gluc-A	Gluc-B
20–25	48 (2,1)	55 (1,9)	50 (2,2)
20–25	125 (1,2)	156 (1,3)	130 (1,2)
20–25	241 (1,5)	265 (1,8)	250 (1,7)
41–45	48 (2,1)	50 (1,9)	49 (2,2)
41–45	125 (1,2)	127 (1,3)	127 (1,2)
41–45	241 (1,5)	238 (1,8)	240 (1,7)
54–61	48 (2,1)	45 (1,9)	47 (2,2)
54–61	125 (1,2)	102 (1,3)	120 (1,2)
54–61	241 (1,5)	220 (1,8)	237 (1,7)

Gluc-A: glucómetro que no ajusta el resultado según hematocrito real-Freestyle Optium Neo (Abbott, Lake, IL, EE.UU.); método electroquímico: Glucosa Deshidrogenasa. Gluc-B: glucómetro que ajusta el resultado según hematocrito real- Nova StatStrip (Nova-Biomedical, Waltham, EE.UU); método electroquímico: Glucosa Oxidasa. CV, variabilidad analítica alrededor de valor medido.

Nuestra opinión coincide con la de los autores en que la mejora en los glucómetros, tanto en análisis como en conectividad a historia electrónica y trazabilidad total, hará que ganen peso en algunos procesos asistenciales, disminuyendo demora en diagnóstico y tratamiento, y reduciendo costes analíticos y clínicos.
